# Current Progress of EMT: A New Direction of Targeted Therapy for Colorectal Cancer with Invasion and Metastasis

**DOI:** 10.3390/biom12121723

**Published:** 2022-11-22

**Authors:** Zhuomin Tan, Wenyan Sun, Ya Li, Xingmeng Jiao, Mingliang Zhu, Junfei Zhang, Chen Qing, Yinnong Jia

**Affiliations:** 1 Department of Pharmaceutical Sciences, School of Pharmaceutical Sciences and Yunnan Key Laboratory of Pharmacology for Natural Products, Kunming Medical University, Kunming 650500, China; 2 College of Chinese Medicine, Yunnan University of Chinese Medicine, Kunming 650500, China

**Keywords:** colorectal cancer, EMT, tumor invasion and metastasis, targeted therapy, signal pathway

## Abstract

Colorectal cancer (CRC) is a common malignant tumor with a high frequency of recurrence and metastasis, which are the major causes of death in patients. The prerequisite for the invasion and metastasis is the strong mobility of CRC cells to transport far away from the original site to the distant organs and tissues, where they settle down and proliferate. It was reported that the epithelial-mesenchymal transition (EMT) is involved in the occurrence and development of various tumors in the entire process of tumor invasion and metastasis. Therefore, as a vital factor for the biological characteristics of tumor cells, EMT markers may serve as prognostic predictors and potential therapeutic targets in CRC. This article mainly reviews the current status of CRC with metastasis, the studies of EMT, the possible relationship of EMT with CRC, as well as the potential targeted therapy.

## 1. Colorectal Cancer

According to the annual report of the World Health Organization (WHO), CRC is the third highest incidence of malignant cancer worldwide, following lung cancer and breast cancer [1]. Although the early screening for diagnosis and treatment of CRC has been continuously improved in recent years, its incidence and mortality rates still remain high [2]. By 2022, the number of newly diagnosed CRC patients is expected to reach 151,030, and the number of deaths is expected to reach 52,580 [3], By 2030, approximately 1.1 million deaths caused by CRC worldwide annually, with 2.2 million new cases, will be reported [4]. From 1990 to 2019, CRC cases in China increased from 105,900 to 607,900 and the standardized incidence rate increased from 12.52 to 30.55 out of 10 million. The number of death increased from 79,300 to 261,800, and the standardized mortality rate increased from 10.18 to 13.86 out of 10 million. Compared with the data from 1990, CRC incidence cases, the standardized incidence rate, the number of death cases, and the standardized mortality rate in China increased by 474.03%, 144.01%, 230.14%, and 36.15%, respectively [5].

### 1.1. CRC Treatment

Currently, CRC treatment may include surgery and non-surgical therapy. Surgery mainly includes new surgical treatment (complete mesocolic excision), abdominal surgery, and palliative surgical treatment [6]. Non-surgical therapy mainly includes radiotherapy, new chemotherapy (fluorouracil drugs [7]), targeted therapy, immunotherapy [6] (immune checkpoint inhibitors, Pembrolizumab and Nivolumab), neoadjuvant therapy (preoperative chemotherapy or radiotherapy) [8], and sandwich therapy (preoperative, intraoperative, and postoperative chemotherapy). Drugs involved in the treatment of CRC include regular chemotherapy drugs, targeted drugs, and biological agents. Fluorouracil was the first recognized drug for the effective treatment of CRC [9]. Later, oxaliplatin and irinotecan were successively approved, and their combination was selected as the first choice for CRC. The occurrence of molecule-targeted drugs, such as the epidermal growth factor receptor (EGFR) inhibitor, cetuximab, brings new promise for CRC treatment.

The 5-year survival rate of patients with early diagnosed CRC is greater than 90%. However, as a result of no obvious symptoms in the early stage and the lack of sufficient effective diagnosis methods, CRC was commonly diagnosed in the advanced stage. In recent years, with the progress of surgery and treatment, the therapeutic efficacy of CRC patients has greatly improved. Unfortunately, the 5-year survival rate of patients with advanced CRC is still very low, this being less than 12% [10].

### 1.2. Invasion and Metastasis of CRC

As described, tumor invasion and metastasis are determined by the transition of the intrinsic properties of tumor cells, especially closely related to the microenvironment [11]. The tumor microenvironment exhibited obvious differences compared with the normal internal environment of the human body in terms of physical and chemical properties, with the characteristics of low PH, low oxygen, and high pressure. Thus, it caused the accumulation of cellular chemokines, growth factors, and immunoinflammatory mediators produced by various proteolytic enzymes, which lead to a series of changes in tumor proliferation, angiogenesis, invasion, and anti-radiation chemotherapy. Stage 0 carcinoma does not invade or grow outside the intestinal wall and, once diagnosed, can be completely removed with an endoscopic polypectomy. At stage 1 and 2, cancer cells invade the colon wall and have not been detected nearby or at different sites. At stage 3, tumors begin to spread to nearby lymph nodes with tumor deposits generated, and at stage 4, the metastasis spread to distant organs [12].

**Figure 1:**
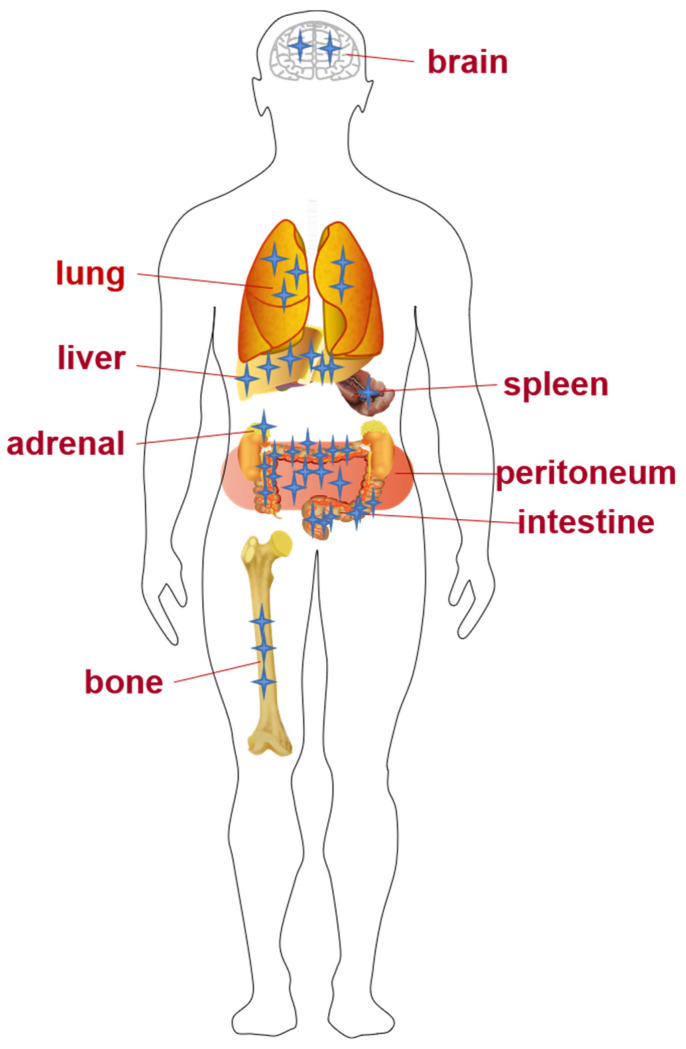
Invasion and metastasis of CRC. The number of asterisks in organs represents the amount of CRC tumor cells localized.

CRC with distant metastasis, one of the main causes of death, was found in about 20% of patients at their first diagnosis [13]. Even worse, 50% to 60% of patients with orthotopic CRC will eventually develop metastases, among which 80% to 90% belong to unresectable liver metastases [14]. Liver is considered to be the most common site of CRC metastasis because most of intestinal mesenteric drainage enters the hepatic portal venous system. Furthermore, the lung was found to be the second most common metastatic site of CRC [2], with this being the case for 10% to 25% of patients. Currently, studies of lung metastases are relatively deficient compared with liver metastases. In addition, in a retrospective study of CRC patients, the peritoneal metastasis rate was between 4% and 13% [15]. Furthermore, bone and brain metastasis of CRC were also reported. As an advanced indication of CRC, bone metastasis represents only 1% to 2% of cases, which is relatively rare clinically, but its incidence has gradually increased in recent years [16], whereas the incidence of brain metastasis is only 0.6% to 4% [17]. A few cases were reported in which CRC metastasized to the adrenal gland [18] and spleen [19] exclusively (Figure 1).

## 2. Epithelial Mesenchymal Transition

**Figure 2:**
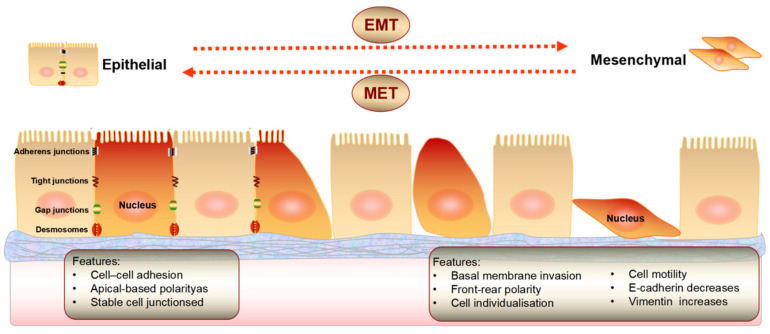
The conversion of epithelial mesenchymal transition (EMT) and mesenchymal epithelial transformation (MET).

EMT is a highly conserved and fundamental process that controls morphological changes during development. EMT is a process in which cells lose polarity and break intercellular junctions (including tight junctions, adhesion junctions, gap junctions, and desmosomes), thus, the integrity of the basement membrane is destroyed, and cells transform from immobile epithelial cells to mobile mesenchymal cells. The intercellular adhesion ability of interstitial cells is decreased, and the mobility ability is enhanced, which can promote the invasion and metastasis of tumor cells (Figure 2). EMT is a reversible process and the opposite direction is named as mesenchymal epithelial transition (MET). EMT and MET are interrelated and interact in their individual biological process in a certain order, in which EMT dominates the early reaction, whereas MET regulates the late stage [20].

The decrease of *E*-cadherin and the increase of vimentin can be considered as symbols of the occurrence of EMT. *E*-cadherins are transmembrane glycoproteins whose extracellular regions can mediate adhesion to neighboring cells [9]. As a cytoskeletal protein in mesenchymal cells, vimentin appears only in the tumor stroma, without showing up in normal epithelial cells [21].

The EMT process involves a synergy of different signal transduction pathways and is also co-regulated by various cytokines and growth factors [22]. In most cases, invasive cells and tumor metastasis at secondary sites are the most common causes of cancer death [23]. Many studies [24,25,26] found that EMT was very important and associated with the invasion and metastasis of CRC, liver cancer, pancreatic cancer, breast cancer, gastric cancer, and other cancers.

## 3. EMT in CRC Invasion and Metastasis

During tumor development, the EMT process of cell is indispensable, and is also considered to be the primary element of the development and metastasis in CRC [27]. Transforming growth factor β (TGF-β) is a multifunctional cytokine expressed in the colon and plays a critical but paradoxical role in CRC. TGF-β is a potent proliferative inhibitor of normal epithelial colon cells, thus acting as a tumor suppressor. However, TGF-β also promotes the invasion and metastasis of CRC at advanced stages. Recently, TGF-β was found to be an important inducer of EMT, regulating EMT through the Smad and non-Smad signaling pathways [5]. Schwitalla et al. [28] demonstrated that p53 suppressed the inflammatory microenvironment associated with NF-κB activation in the advanced stage of disease, and that the loss of the p53 gene and related NF-κB pathway activation ultimately induced EMT in tumor cells, which might be a necessary event to regulate colon tumor progression.

It was also found that EMT directly or indirectly downregulated the expression of E-cadherin to regulate the development of CRC, mainly through three core transcription factors: (1) the transcription factors of the Snail zinc finger protein family (Snail1 and Slug); (2) the E-box binding zinc finger protein homology protein family (ZEB1 and ZEB2) [29]; and (3) the basic helix-ring-helix family of transcription factors (Twist1, Twist2, and E12). Ahmadiankia et al. [30] found a significant correlation between the expression of Twist (OR, 1.46; 95% confidence interval [CI], 1.03–2.09), Slug (OR, 3.43; 95%CI, 1.98–5.93) and ZEB2 (OR, 2.42; 95%CI, 1.09–5.40) with distant transfer, showing that the expression of Twist, Slug, Snail1, ZEB1, and ZEB2 was associated with poor CRC survival. In addition, 85% of tumor specimens exhibited a moderate-to-intense expression of Twist, indicating that Twist may be an important prognostic marker for CRC. Bolós et al. [31] found that Slug could inhibit E-cadherin promoter activity by 50% at 240ng by comparing the binding ability of Snail1 and Slug to E-cadherin promoter. Aigner et al. [32] investigated the potential ZEB1 targets involved in epithelial cell differentiation through RNA-mediated ZEB gene silencing, and they found that loss of ZEB1 also induced the expression of *E*-cadherin and many critical genes related to epithelial cell adhesion and differentiation, such as the *E*-cadherin-related gene CDH1. The NF-κB and PI3K/AKT signaling pathways, which play an important role in the regulation of EMT and tumorigenesis, also serve as important regulators of ZEB1 transcription.

After EMT occurred, malignant tumor cells acquire the ability of invasion and metastasis, which can pass through the lymph tract, blood vessel, or body cavity to grow at other sites [33] where new metastatic lesions are formed by proliferation. It is an important biological process in the tumor invasion of surrounding tissues and metastasis of distant organs [6]. EMT is coordinated by relevant factors that transduce features that are critical for the malignant progression of cancer cells, including tumor-initiating properties, motility, and transmission ability [34]. Many studies have shown a close relationship between EMT and the acquisition of stem cell-like properties, which are characterized by tumor initiation, self-renewal, metastasis, and chemotherapy resistance [35]. EMT promotes tumor metastasis partially by the enhancement of cell motility [36]. Three protein complexes (Par, Crumbs, and Scribble) maintaining apical-basal polarity in epithelial cells [37] are regulated by the EMT-inducible genes. *E*-cadherin downregulation and *N*-cadherin up-regulation are important steps in most EMT processes. *E*-cadherin, an essential cell-to-cell adhesion protein, forms epithelial cell sheets and maintains cell quiescence [38]. In contrast, the increased expression of *N*-cadherin is generally observed in migrating neurons and mesenchymal cells to enhance motility and spread.

In addition to being mobile and aggressive, cells undergoing EMT also gained resistance to drugs. The overexpression of non-coding regions of EMT is sufficient in cells with ineffective protein coding of Snail or ZEB1 to increase treatment resistance [39]. TGF signaling was found to play a key role in HCT116 human CRC cells with doxorubicin-resistance [40]. TGF pathway inhibition can reverse EMT and thus, enhance cancer cell sensitivity to doxorubicin, proposing potential novel therapeutic strategies to overcome chemoresistance in CRC. EMT-inducing transcriptional factors such as ZEB1, Snail, and Slug promoted resistance to oxaliplatin-based and cisplatin-based chemotherapy in cancer. Hoshino et al. [41] found that Snail could improve the resistance to 5-fluorouracil and increased EMT in CRC cell lines. In addition to developing resistance to chemotherapy, tumor cells demonstrated resistance to immunotherapies by EMT [28]. TGF plays a dual regulatory role in CRC as a key and polymorphic regulator of the immune response that is required for immunosuppression in regulating T cell formation and reducing the cytotoxicity of NK cells [42]. It is unclear whether immunosuppression is a direct result of the EMT progress or develops through the mediation of other yet uncharacterized signaling pathways. Whether the EMT-inducing transcription factor directly stimulates the expression of immunomodulatory markers, and how these markers confer tumor cell resistance to various checkpoint blockers in CRC, remains to be determined. Overall, the occurrence of EMT greatly increases the difficulty and expense of treatment for metastatic CRC.

Brabletz et al. [43] used *E*-cadherin and catenin to explore the phenotype transition process and its potential regulatory force during metastasis formation. In their study, MET was first detected in metastasis, and in most cases the same differentiation as the primary tumor occurred. Thus, they proposed a well-differentiated tumor progression model including the epithelial and mesenchymal phenotype transition. EMT as an invasion frontier can induces tumor cell dedifferentiation, subsequent re-differentiation, restoration of epithelial characteristics, and basic survival ability process, suggesting that EMT is a transient process [44]. Under the dynamic regulation of tumor environmental factors, the whole process of invasion and metastasis is very inefficient, therefore, only a small fraction of cancer cells left from the primary tumor can successfully form visible metastases [37]. In this process, tumor cells need to undergo the reverse process, MET, and EMT needs to be transformed into MET to successfully colonize at a distant organ. In conclusion, tumor invasion and metastasis and EMT are interactive and interrelated. Heterogeneous populations with different degrees of EMT can be observed within a wide range of cancers.

Notably, the conclusion of EMT cannot be fully applied to the invasion and metastasis of CRC. The important marker of EMT, vimentin, is not equally expressed in different cell lines of the same tumor [5]. In detail, vimentin is highly expressed in the CRC cell line SW480 but undetected in other cell lines undergoing EMT such as HT-29, SW948, and RKO. In addition, some studies suggested that EMT was not required for cancer metastasis [43,45,46]. Many primary epithelial tumors that grow in an epithelial pattern with completed differentiation can also recapitulate the basic morphology of the original tissue after metastasis, without the evidence of EMT [46]. Some well-differentiated colorectal adenocarcinomas that grow in tubular structures retain the epithelial phenotype and could metastasize [43].

## 4. Targeted Therapy Associated with EMT

CRC is a highly heterogeneous disease with a variety of molecular subtypes and genetic variation. Only a small number of patients with CRC metastasis are suitable for surgical treatment for reasons such as too large organ metastasis, too close to large blood vessels, and multiple metastases. For patients with CRC at an advanced stage, novel drugs need to be developed by the study of the metastasis process so that patients can obtain individualized treatment to further improve the survival rate and quality of survival. Targeted therapy is a current research hotspot with the advantages of better efficacy and less adverse effects.

### 4.1. EMT and Genes

The focus on molecular biomarkers has switched from simple prognostic predictors to potential therapeutic targets. More than half of CRC cases exhibited mutations in the KRAS and BRAF genes [47]. Then, drug targets were developed, such as the BRAF inhibitor Encorafenib and the KRAS inhibitor Adagrasib. Oncogene addiction is a new strategy for developing tumor targeted drugs, which induces oncogene-dependent cell death with the termination of oncogene-induced signaling. Yang et al. [48] selected multiple EMT-related genes from the molecular signature information database and analyzed the correlation with CRC prognosis, metastasis, drug efficacy, and immunity by bioinformatics, and finally found nine EMT-related genes, including FGF8, NOG, PHLDB2, SIX2, SNAI1, TBX5, TIAM1, TWIST1, and TCF15. Targeting these nine prognostic-related genes can regulate CRC metastasis and immunity to benefit the prognosis and improve the drug sensitivity of CRC, which is expected to be a new target for future targeted therapy and immunotherapy.

The significantly different expression of RNA in CRC tumors and normal tissues suggests that RNA may be used as a molecular marker for the non-invasive diagnosis and prognosis of CRC. On the other hand, RNA interference and miRNA silencing provide new directions for the treatment [49]. Some miRNA and non-coding RNA can regulate key EMT genes to affect the EMT program. After examining the expression profile of miRNAs in CRC tissues and comparing it with normal tissues, it was found by [50] that the expression of miR-21, miR-31, and miR-135b were significantly upregulated, whereas miR-34a, let-7a, miR-145, and miR-143 were lowly expressed. During EMT in CRC, long non-coding RNA (lncRNA) [51] include LINC01133, SLC25A25-AS1, and lncRNA-CTD903 were down-regulated, whereas H19, HOTAIR, MALAT1, SPRY4-IT1, TUG1, CCAT1, and lncRNA-ATB were up-regulated.

#### 4.1.1. EMT and miRNA

Studies have shown that miRNA is closely related to the occurrence, invasion, and metastasis of CRC, which regulate the expression of genes as tumor suppressors or inducers. For example, miRNA-375 acts as a tumor suppressor to inhibit the invasion and migration of CRC by inhibiting EMT [52]. In the study [53] of miR-145 and its inhibitors, it was shown that miR-145 overexpression increased E-cadherin and reduced vimentin significantly, whereas miR-145 inhibition brought the opposite effect. The invasion and migration of CRC were inhibited by miR-145, which negatively regulated Twist1 level at the transcriptional level and inhibited EMT. The miR-200 family including miR-200a, miR-200b, miR-200c, miR-141, and miR-429 were significantly downregulated in cells undergoing EMT, and it has been suggested that the forced expression of the miR-200 family is sufficient to prevent TGF-induced EMT. Rokavec et al. [54] explored the role of the IL-6R/STAT3/miR-34a feedback loop in promoting EMT-mediated invasion and metastasis of CRC, and p53 acted as tumor suppression by inducing miR-34a activation and disrupting this feedback loop. In addition, miRNA also affects the sensitivity of tumors to drugs. Jiang Li et al. [55] confirmed that miR-489 can stimulate chemoresistance in human breast cancer by regulating the Smad3-mediated EMT in doxorubicin-resistant-cells. Moreover, regulating CRC invasion and metastasis, miR-489 acts as a tumor suppressor gene by interfering drug resistance, making miR-489 a potential therapeutic target for CRC patients [50].

#### 4.1.2. EMT and lncRNA

Although lncRNA is less characterized than small non-coding microRNA [56], lncRNA may serve as potential therapeutic targets and promising candidates for future cancer diagnosis and therapy [57]. A better understanding of how lncRNA regulates EMT progression at different molecular levels [58] would promote the development of innovative therapeutic strategies. Guo et al. [56] showed that the expression of BRAF-activated lncRNA (BANCR) was significantly higher in tumor tissues than that in adjacent normal tissues. BANCR downregulation in HCT116 cells was found to be associated with the upregulation of E-cadherin expression and the downregulation of vimentin at the mRNA and protein levels, opposite to that in BANCR-overexpressing Caco2 cells. Moreover, the MEK/ERK signaling pathway inhibitor (U0126) inhibited cell migration induced by BANCR overexpression to promote vimentin upregulation and E-cadherin downregulation, suggesting that BANCR induces EMT through a MEK/ERK-dependent mechanism to promote CRC migration. lncRNA affects EMT progression either directly or indirectly by targeting multiple EMT markers that play a key role in all tumor types, thereby affecting tumor development and progression, as well as drug resistance. Different lncRNAs play different roles in EMT [56]. For example, SNHG15 promotes EMT by elevating Slug protein expression, whereas LINC01133 binds to SRSF6 and blocks its key domains to inhibit EMT. Zhang et al. [59] found that lncRNA H19 expression was significantly upregulated in primary and metastatic tumors and correlated with poor prognosis in CRC, suggesting that H19 may be a potential biomarker to predict prognosis as well as a therapeutic strategy for CRC. The identification of biomarkers allows for a precise diagnosis and specific therapy. In this case, prognostic indicators determine treatment intensity and decrease the risk of tumor recurrence or progression. Thus, individual markers associated with EMT can serve as predictors of treatment and survival outcomes in CRC patients.

### 4.2. EMT and Proteins

The traditional view of gene regulation in biology focuses on the gene-encoded proteins in order of DNA-mRNA-proteins [60]. Most of the recently approved molecule-targeted cancer drugs are specific for the oncoproteins encoded by somatic mutant genes. It has been shown that heat shock protein 90 (Hsp90) also binds to and stabilizes Snail, and that Hsp90 and its ligand proteins such as Akt and c-Src play important roles in cancer development undergoing EMT by regulating various signaling proteins, which is a valuable target for cancer therapy. The AHA1 gene was identified by Kim et al. [61] as an activator of the ATP enzyme activity of Hsp90. Heat shock protein and AHA1 genes were significantly more highly expressed in 105 CRC samples compared with paired normal tissues. In the overexpressed or knocked down of AHA1 in CRC cells, various molecules such as E-cadherins responded obviously to this change. AHA1 expression can increase the activity of Hsp90 ligand proteins and phosphorylate key signaling proteins to affect the benefits of Hsp90 inhibitors, demonstrating that the targeted disruption of the Hsp90-AHA1 complex increases sensitivity to Hsp90 inhibitors in cancer cells. AHA1 may serve as a potential prognostic marker associated with lymph node and metastasis. The tetraspanin family member, claudin [62], is a dynamic protein that helps to regulate cell functions such as cell proliferation, migration, and differentiation. Cellular localization of claudin-1 protein may determine the fate of CRC patients, which upregulates the suppressor ZEB1, reduces E-cadherin expression, and increases its invasive activity in CRC cells, a pathway that is associated with CRC progression and patient survival. Cancer testis (CT) antigen is one of the hotspots of developing therapeutic targets, with A-kinase anchor protein 4 (AKAP4) being seen as a novel CT antigen. Jagadish et al. [63] analyzed AKAP4 expression and its potential role in cell migration and invasion. They found that the knockdown of this gene led to the downregulation of EMT markers and the invasion molecular matrix metalloproteinases MMP2, MMP3, and MMP9. By removal of the AKAP4 protein, migration capacity was reduced by 71.17% and 68.28%, respectively, and the invasion capacity was reduced by 72.35% and 67.52%, respectively. Liu H et al. [64] found that the traditional chemotherapeutic drug vinorelbine, with a unique affinity for mitotic tubulin, significantly upregulated E-cadherin expression and downregulated *N*-cadherin and vimentin expression, to inhibit its migration, which sparked new ideas for cancer treatment.

### 4.3. EMT and the Signaling Pathways

**Figure 3:**
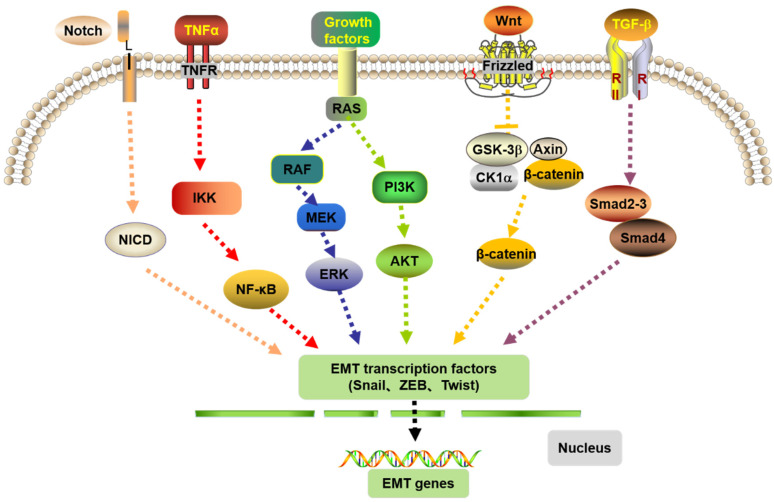
EMT-transcriptional factors activated by extracellular and intracellular pathways to regulate EMT. From left to right: Notch, NF-ĸB, RAF/ERK, PI3K/Akt, Wnt/β-catenin, TGF-β.

Many pathways are involved in the development of CRC (Figure 3), among which Notch signaling pathway is activated by the interaction between the Notch receptor and ligand, which increases the expression of EMT transcription factors Snail, Slug, and ZEB. TNF-α can stimulate the downstream nuclear factor NF-κB to transfer to the nucleus and regulate the expression of Snail and Twist genes to mediate EMT. The RAS signaling pathway can regulate EMT through various mechanisms, such as stabilizing Snail through PI3K/Akt signaling and regulating transcription factors such as Twist, Snail, and Slug via RAF/ERK signaling to promote EMT. In addition, the Wnt signaling pathway is also an important pathway in regulating EMT. The classical Wnt pathway uses β-catenin as the key node to regulate the downstream pathway. The β-catenin is inhibited and cannot be phosphorylated, which leads to its accumulation in the cytosol and, finally, its migration to the nucleus to activate EMT transcription factors. In TGF-β signaling pathway, Smad2, 3, and 4 are activated and form complexes under the action of TGF-β activation and finally interact with other transcription factors to induce the occurrence of EMT gene transcription.

Dickkopf-1 (DKK1) is an inhibitor of the Wnt signaling pathway that can inhibit CRC progression by inhibiting EMT [65]. Canel et al. [66] found that the Src/FAK signaling prevents the movement of tumor cells in vitro and in vivo, and the FAK inhibitor PF-562271 inhibited tumor cell spread and metastasis. Polysaccharide-K (PSK) was found to modulate the biological activities of TGF-1 and TGF-2 [67]. Thus inhibiting the EMT process, the randomized clinical experiments also showed that PSK plays an effective role in the adjuvant treatment of CRC [68]. Hou [37] revealed the unknown mechanism of LHPP as a tumor suppressor in CRC metastasis, where E-cadherin was significantly increased with LHPP overexpression. As a key mediator of the classical Smad-signaling TGF-β/Smad3 phosphorylation, LHPP specifically inhibited EMT, providing new perspectives for the treatment of CRC.

### 4.4. EMT and the Correlated Factors

The invasion and metastasis-related factors are crucial points in CRC treatment and drug development. In the study of the mechanism of basement membrane turnover in malignant CRC progression, Spaderna et al. [44] proposed that the progression of basement membrane loss and the differentiation of epithelial cell phenotype upon ZEB1 knockdown is similar to MET, and targeting relevant EMT-related transcriptional repressors in ZEB1 or other tumors may be a choice with therapeutic potential to prevent malignant metastatic progression. Studies by Hu et al. [69] verified that HDAC2 acted as a metastasis suppressor in CRC by inhibiting the expression of EMT, H19, and MMP14. Although the development of anticancer drugs targeting HDACs highly expressed in many tumors has been conducted for many years, clinical trials have shown that the single application of HDAC inhibitors are ineffective in patients with solid tumors, including CRC. Therefore, more studies of HDAC inhibitors in solid tumors need to be carried out. As an EMT-related factor [25], cMET promotes EMT in CRC by inhibiting RKIP expression. In this case, patients with c-MET downregulation and RKIP upregulation showed the longest overall survival and progression-free survival time. Thus, targeting RKIP to treat cMET-induced CRC metastasis is a new and feasible idea. At present, c-Met inhibitors are approved for clinical practice, such as HLX55 and SHR-A1403, and enhance the confidence in the innovation and development of cMET-related drugs [70].

### 4.5. EMT and MET

The downregulation of EMT inducers and the subsequent MET are necessary for metastasis and colonization at a large scale [71]. The induction of differentiation and targeting EMT alone may lead to the reversed effect through proliferation of disseminated cells, therefore, targeting therapies in different cell cycles needs to be combined. Furthermore, the maintenance of tumor cell quiescence by MET inhibition and directly targeting the stem cell phenotype may be promising strategies, regardless of the tumor location.

In order to solve a large number of problems derived from the EMT during the invasion and metastasis of CRC, researchers have developed many relevant drugs to regulate the expression of invasion and metastasis-related factors and then inhibit the EMT process, which is also one of the clinical strategies to reduce tumor invasion and metastasis, as well as chemoresistance. Widely used bevacizumab can bind specifically to vascular endothelial growth factor (VEGFR) to inhibit tumor metastasis by reducing microvascular growth. As a current systemic therapy, regorafenib [72] is an oral multi-kinase inhibitor targeting the VEGFR and the tumor microenvironment. Tyrosine kinase inhibitors have been shown to be one of the effective drugs in tumor treatment, not only promoting the stromal to epithelial phenotype [14], but also inhibiting tumor invasion and metastasis. Molecular targeted drugs are also used in some cases of clinical treatment failure, for example, cetuzumab is used to treat the clinical treatment failure of oxaliplatin, and as a neoadjuvant therapy can increase the survival rate of surgical resection by 50%. Cetuximab is a monoclonal antibody that can specifically bind epidermal growth factor receptor (EGFR) and competitively inhibit EGF and other ligands with the receptor blocking the intracellular signal transduction pathway, thus inhibiting the proliferation of cancer cells, inducing the apoptosis of cancer cells, and reducing MMP and VEGF production. Another similar drug is panitumumab. Identifying powerful treatment targets in various cancer types is a high priority [73], and great efforts have led to the successfully targeting of somatic-altered oncoproteins. The possible epigenetic changes caused by the treatment may induce drug resistance in tumor cells, especially during monotherapy [73], thus, the re-expression of silenced tumor suppressor genes may re-sensitize the tumor to treatment [74]. Developing innovative therapeutic agents for newly-discovered cancer targets may provide more patients with effective targeted therapy and also supply complementary agents for combination treatment regimens.

## 5. Conclusions

In the development of most CRC, the inevitable processes are the invasion and metastasis of tumor cells and EMT. These two processes are complementary with multiple signaling pathways and factors. The EMT-related factors and drugs for targeted therapy of CRC were summarized as Table 1. Further in-depth research has found that EMT is not only correlated with the invasion and metastasis of the tumor cells, but can also endow the tumor cells with the characteristics of stem cells as well as lead to the occurrence of cell drug resistance. Therefore, cancer cells with an EMT phenotype not only possess mesenchymal properties, but also exhibit more aggressive behaviors, including resistance to the drug, stress, apoptosis, inhibition of senescence, immune evasion, and acquisition of stem cell-like features [75]. Studies have also found that the tumor microenvironment and autophagy are associated with the EMT of the tumor. Tumor microenvironment-related factors include inflammation, immune cells, tumor-associated fibroblasts, extracellular matrix, signaling molecules, high acidity, and low oxygen. For example, the inflammatory in tumor microenvironment can cause the EMT of the tumor cell phenotype [76]. At the same time, EMT is initiated by hypoxia induction and inflammatory factors in the tumor microenvironment, and the occurrence of EMT also involves the tumor microenvironment. Thus, EMT and tumor microenvironment interact and influence each other to promote tumor metastasis [77]. In addition, a close relationship has been established between autophagy and the development of EMT as a result of autophagy participating in tumor cell invasion and metastasis [78]. The increase of autophagosome in tumor cells plays an important role in developing EMT, which promotes tumor resistance to immune killing, by escaping from cellular immune surveillance [79]. Therefore, a more detailed study of EMT mechanisms would facilitate the development of EMT as an effective new target for tumor treatment, and the diversity of new types of cancer targets provides options for all patients.

## Figures and Tables

**Table 1:** EMT-related factors and drugs for targeted therapy.

				References
EMT	Regulatory proteins	*E*-cadherin		[9]
		Vimentin		
		*N*-cadherin		
	Transcription factor	Snail1		[31]
		Slug		
		ZEB1		
		ZEB2		
		Twist1		
		Twist2		
		E12		
	Drug development	Molecular targeted drugs chemotherapy drugs	5-fluorouracil	[43]
			Regorafenib	[73]
			Bevacizumab	[14]
			Cetuximab	
			Panitumumab	
		Molecular biomarkers	Encorafenib	[49]
			Adagrasib	
		miRNA	miRNA-375	[54]
			miR-145	[55]
			miR-34a	[56]
			miR-200	
			miR-489	[57]
		lncRNA	BANCR	[58]
			SNHG15	
			LINC01133	
			H19	[60]
		Protein	Hsp90	[62]
			AHA1	
			Claudin-1	[63]
			AKAP4	[64]
			Navelbine	[65]
		Inhibitors of Wnt signaling pathway	DKK1	[66]
		Inhibitors of Src/FAK signaling pathway	PF-562, 271	[66]
		Inhibitors of TGF-β signaling pathway	PSK	[68]
			LHPP	[39]
		Targeted RKIP	HLX55, SHR-A1403	[71]

## Data Availability

Not applicable.

## Abbreviations

WHO	World Health Organization
CRC	Colorectal cancer
EMT	epithelial-mesenchymal transition
MET	mesenchymal-epithelial transition
TGF-β	transforming growth factor-β
Smad	drosophila mothers against decapentaplegic
Wnt	Wingless-type
*E*-cadherin	epithelial cadherin
STAT3	Signal transducer and activator of transcription 3
NF-κB	Notch and nuclear factor-κB
*PI3K*	phosphatidylinositol 3-kinase
AKT	protein kinase B
*mTOR*	mammalian target of rapamycin
MAPK	mitogen-activated protein kinase
FAK	Focal Adhesion Kinase
EGF	epidermal growth factor
FGF	Fibroblast Growth Factor
VEGF	vascular endothelial growth factor
ZEB	zinc-finger E-box-binding
c-Met	c-Mesenchymal-epithelial transition factor
*ERK*	extracellular signal-regulated protein kinase
miR	micro RNA
let-7a	let-7amimics
HDACs	histone deacetylases
RKIP	raf kinase inhibitory protein
DKK1	Dickkopf-1
PSK	polysaccharide-K
LHPP	Phospholysine phosphohistidine inorganic pyrophosphate phos phatase
